# Children’s age matters: Parental burnout in Chilean families during the COVID-19 pandemic

**DOI:** 10.3389/fpsyg.2022.946705

**Published:** 2022-09-22

**Authors:** Carolina Panesso Giraldo, María P. Santelices, Daniela Oyarce, Eduardo Franco Chalco, María J. Escobar

**Affiliations:** ^1^Center for Social and Cognitive Neuroscience (CSCN) School of Psychology, Adolfo Ibáñez University, Santiago, Chile; ^2^Escuela de Psicología, Facultad de Ciencias Sociales, Pontificia Universidad Católica de Chile, Santiago, Chile; ^3^Facultad de Ciencias Sociales y Comunicaciones, Universidad de Santo Tomás, Santiago, Chile; ^4^Postgraduate School, Universidad María Auxiliadora, Lima, Peru

**Keywords:** parental burnout, preschoolers, COVID-19 pandemic, gender inequalities, coparental cooperation

## Abstract

For families all over the world, going through a pandemic has presented a number of challenges. In particular, social distancing measures involving the closure of schools and day care centers, as well as increasing work hours at home, made parents face very demanding situations. However, we know little about whether parents’ burnout levels are influenced by the age of their children. This study sought to determine whether levels of parental burnout (PB) are higher in families with at least one child under the age of four than in families with older children (5 to 18 years). The second goal was to explore whether having children under 4 years of age moderates the relationship between parental cooperation and PB. A cross-sectional study was conducted with a sample of 651 participants (525 mothers and 126 fathers) since May 18th until August 27th, 2020. The main results showed that child age is a predictor of PB. Besides, having a child aged 0–4 years old moderates the relationship between parental cooperation and PB. Finally, it was found that in cases where there was at least one child under 4 years of age in the family, with one of the partners who worked remotely, the respondent’s PB rose by 7.9 points. The implications of these results with respect to the consideration of children’s ages in the different parental scenarios were discussed.

## Introduction

The COVID-19 pandemic status declared in 2020 by the World Health Organization revolutionized the world by introducing effects on its inhabitants’ mental and physical health (Akintunde et al., 2021). While the impact on physical health is clear, the effects on mental health in adults and children continue to be studied, showing an increase in prevailing stress responses (Liu and Doan, 2020; Pfefferbaum and North, 2020). Families are no strangers to this reality. Due to the restrictions imposed to reduce infection rates parents had to assume different roles at home (domestic, care, school, work, recreational). The closure of schools and daycare facilities limited the support parents previously counted on, increasing demands and stress responses, which has impacted the quality of care and undoubtedly strained the family’s emotional climate (Adams et al., 2021) putting the focus on parents mental health as well as on children (Mikolajczak and Roskam, 2020).

The home became the school for children and the workplace for adults, diluting the separation of spaces and thus modifying the previously available routines by reinforcing gender roles traditionally assigned to women, such as domestic work and childcare (Hennekam and Shymko, 2020). In Chile, this overdemand for activities has impacted more mothers than fathers. Mothers have increased the hours devoted to caring duties of children under 14 years of age by 14 h per week more than men, and 9 h per week more to work on domestic tasks (Goverment of Chile, 2020). This situation also reflects how women assume the care of children and household activities in Chile, having more significant risks of perceiving stress responses (Pérez and Santelices, 2016).

It is important to consider that before the pandemic, working outside home was considered a protective factor for mothers against depressive symptoms, parental stress (Pérez and Santelices, 2016) and parental burnout (Mikolajczak et al., 2018a). In addition, the pandemic affected the market share women had already achieved, a phenomenon which also affected men, but at much lower rates than the former ones. The closure of daycares, preschools and schools, added to the multiple domestic and care activities, prevented women from looking for work and recovering their labor status (INE, 2020). Prior studies to the pandemic had already proved that mothers in Chile have been significantly exposed to greater and deeper parental burnout in comparison to fathers (Pérez-Díaz and Oyarce Cádiz, 2020). The pandemic confinement in addition to the reduction of parenting support networks, such as caregivers and extended family, were key factors to trigger a syndrome recently described as “Parental Burnout (PB).”

PB occurs in parents exposed to excessive stress, and who do not count with enough resources to make up for the effects of this syndrome (Mikolajczak and Roskam, 2018). Therefore, it can affect any parent who accumulates more risks than resources for too long, as was the case during the COVID-19 pandemic. In Chile, parental stress (which has been the most studied concept) tends to affect mothers with lower educational levels, with more than one child, and with little parental and social support (Olhaberry and Farkas, 2012; Pérez and Santelices, 2016; Pérez-Díaz and Oyarce Cádiz, 2020). Parental stress is conceptualized as the stress provoked by the specific demands of parenting (Abidin and Brunner, 1995; Louie et al., 2017), characterized by the presence of acute stressors. This type of stress, in turn, may be related to other types within the family context, such as those caused by social and economic demographic factors (Abidin, 1990 cited by Sandoval et al., 2022), while PB represents chronic parental stress over time, what leads to a unique syndrome where parents feel physically and psychologically overwhelmed, where emotional estrangement arises from children, as well as the sense of failing at meeting their parental role, which turns into guilt and the contrast between the kind of parent they would like to be, and the ones they have become. In this syndrome, unlike parental stress, the demographic, social, and economical context are not enough data to effectively foresee PB. Other factors, such as parents personalities and their main features (perfectionism, neuroticism, attachment issues, and low emotional intelligence levels), parenting factors (lack of positive parenting, low levels of self-esteem in the regard of their parental role, more restrictive parents), and issues within the family itself, what affects the way families work (low levels of parental support or low co-parenting, permanent argument, disagreements in the couple regarding parenting, low satisfaction with the partner, and family disorganization, among others; Mikolajczak et al., 2018a).

Considering that within the factors of family functioning, parental support or co-parenting are elements that allow us to foresee PB (Roskam et al., 2018), and considering that parent cooperation refers to how mothers and fathers work together to perform the tasks of parenting (Feinberg, 2003), it is relevant to inquire about the collaboration that participants receive from their partners regarding the care and upbringing of their children. Taking into consideration that during the COVID-19 confinement, domestic and care activities have been primarily performed by women, it is congruent that they are the most affected, without considering that the exercise of co-parenting in Chile is still in its early stages (Pérez-Díaz and Oyarce Cádiz, 2020), which further increases risks of PB on mothers.

On the other hand, the age of children is a variable that, although it does not play a predominant role at PB’s arisal, if it is present, it contributes to trigger one of the main symptoms of PB: physical exhaustion (Mikolajczak et al., 2018b). This could be mainly linked to the demands of younger children, given that they require more attention from their parents, which also involves a greater quantity and quality of organization from parents in order to respond to their children’s needs and care. Therefore, in the Chilean context where co-parenting is incipient, the age of children as a tool to predict PB is considered key in this study. A Finnish study (Sorkkila and Aunola, 2022) conducted during the pandemic found that having children under the age of 10 and spending more time with them is a particular risk factor for PB. However, this study only considered children under the age of 10, whereas in ours we seek to be more specific in the regard of age ranges that may be directly proportional to the demands of parenting and child development, from newborns to 18 years of age. We hypothesize that the younger the age the greater the challenges for parents in what relates to care and parenting. This is key since in another research developed in pandemic context in Finland (Upadyaya and Salmela-Aro, 2021) where they used latent profile analysis of PB dimensions, it was found that parents who reported their children had more challenging characteristics, belonged to high burnout and emotional distancing profiles. The main goal of this study was to determine whether PB levels are higher in families with at least one child under the age of four than in families with older children (5 to 18 years), considering the described background and current health status. The second goal was to explore whether having children under 4 years of age moderates the relationship between parental cooperation and PB.

## Materials and methods

### Procedure

This study is part of the International Investigation of Parental Burnout (IIPB) Consortium, being part of its second data collection version worldwide. It has the approval of the Ethics Committee of three universities, Adolfo Ibáñez University (23/2020), Pontifical Catholic University of Chile (200425001), and University of Tilburg in the Netherlands (EC-2018.13). A cross-sectional and correlational study was conducted where the sample was called through different social networks (Instagram and Facebook) and websites linked to parenting, childhood and psychology research, as well as the personal accounts of the researchers of the study, creating a snowball effect. The data was collected by using a self-report questionnaire completed online through the Google Forms platform, after registration of informed consent that described the characteristics of the study and information about the confidentiality and anonymity of the data. There were no payments for the participants of this study. The distribution allowed the survey to be answered in all regions of Chile. However, data was mainly collected from the Metropolitan Region.

### Participants

The total sample consisted of *N* = 719 participants who live in Chile. Participants who had no children living in the household (*N* = 5) together with those who had children older than 19 years old (*N* = 63) were excluded. For objective 1, participants with no children in the household (*N* = 5) and those with children over 19 years of age (*N* = 63) were excluded, consolidating a sample of 651 participants (*N* = 651, 525 mothers and 126 fathers). As for the second objective, parental cooperation is an important variable and implies the existence of a couple, therefore, participants who declared they did not live as a couple (*N* = 90) were excluded too, consolidating a sample of 561 participants (*N* = 561, 439 mothers and 122 fathers).

The samples selected for both objectives are similar (see Appendix 1). Participants are around 40 years old and have two children in the household. Those who responded are mainly mothers (80.65%), from two-parent families (> 86%) with household income above US$ 2,500 (60%), and completed undergraduate and postgraduate studies (approximately 80%).

### Instruments

The data were collected through the International Investigation of Parental Burnout (IIPB) Consortium (for more information about the IIPB Consortium, see Roskam et al., 2021). The common IIPB protocol included several measures designed to address different research questions and goals (e.g., comparing the prevalence of parental burnout across countries; investigating the relations between parental burnout and perceived-ideal parental self-discrepancies; examining the contribution of different parental duties to parental burnout). As these questions are too diverse to be addressed in the same article, we describe below the measures used in the current paper only. The full IIPB protocol is available on Open Science Framework (OSF) at: https://osf.io/94w7u/?view_only=a6cf12803887476cb5e7f17cfb8b5ca2.

#### Sociodemographic questionnaire

This questionnaire included sex, age, educational level, type of family (two-parent, single-parent, reconstituted, same-sex parent, multigenerational, polygamous), type of restriction as a measure against the COVID-19 pandemic, monthly income, remote work and overload of household chores. The number of children and their age was also asked and grouped into age ranges, as follows:

*- How many children (of those living in your household) are between 0 and 4 years old? (example 1; write only the number).*

*- How many children (of those living in your household) are between 5 and 9 years old? (example 2; write only the number).*

*- How many children (of those living in your household) are between the ages of 10 and 14? (example 2; write only the number).*

*- How many children (of those living in your household) are between the ages of 15 and 18? (example 2; write only the number).*

*- How many children (of those living in your household) are over the age of 19? (example 2; write only the number).*

#### Parental burnout assessment PBA

The PBA questionnaire is composed of 23 items such as *“I feel as if I’ve lost my direction as a dad/mom”* on a frequency scale ranging from “never” (0 points) to “every day” (6 points) and containing the scales of Exhaustion (*α* = 0.93), Contrast (*α* = 0.94), Saturation (*α* = 0.91) and Emotional distancing (*α* = 0.77). It is a validated instrument to measure burnout or parental exhaustion, presenting high reliability (*α* = 0.96). For this study we used the global measure of Parental Burnout assessment scale (Roskam et al., 2018).

#### Coparental cooperation scale

This scale is part of the Coparentality Inventory designed by Teubert and Pinquart (2011), which evaluates the support, respect, and cooperation participants receive from their partners or other people living in the home regarding the care and upbringing of their children. It consists of four items such as *“If there is a problem with our child(ren), we search for a solution together”* scored from 1 to 5: (1) completely false and (5) completely true. This scale has good internal consistency (*α* = 0.89) with high correlations between each item and the total scale (*α* = 0.74 and 0.87, respectively; Teubert and Pinquart, 2011).

### Statistical analysis

As a first descriptive step statistics were estimated for the population studied. In this step a small percentage of missing data was identified (4.5%), although the source of this missingness was non-systematic or MCAR (Graham et al., 2012). To address this problem, multiple imputation with predictive mean matching (PPM) was used for latter analyses. To address the first study objective, a regression analysis was performed on the Parental Burnout as a dependent variable, and child’s age as a predictor one, using the parent’s gender, where the respondent or the respondent’s partner worked remotely with individual overload worked as covariates. In the same way, to address the second study objective, a regression analysis was performed on Parental Burnout and child age, with co-parent cooperation as predictor variables. Newly, parents’ gender, the factor of the respondent or their partner worked remotely and individual overload were included as covariates. In this model, an interaction term between child age and co-parent cooperation was also added to assess whether the presence of younger children at home moderates the relationship between co-parent cooperation and PB controlled by covariates (Cohen et al., 2013). Finally, regression assumptions were analyzed such as: normality, homogeneity of variance and multicollinearity (Cohen et al., 2013), all of them being met. All the analyses were held on RStudio software, version 1.3.1073 (RStudio Team, 2020).

## Results

### Descriptive statistics

The descriptive statistics for the Parental Burnout Scale (PBA) taking into account objective 1 (*N* = 651) show average scores of 34.58 points in the total sample (*DS* = 33.05), with scores ranging from 0 to 137. In objective 2 (*N* = 561), the PBA scale shows average scores of 34.58 points in the total sample (*DS* = 33.11) with scores between 0 and 137, and the co-parent cooperation scale shows average scores of 17.38 (*DS* = 3.76) with scores ranging from 4 to 20 (see Tables 1, 2).

**Table 1:** Descriptive of PB for objective 1.

Variable	N	Mean	SD	Min	Max
Burnout Parental	651	34.58	33.05	0	137

**Table 2:** Descriptive of PB and COPs for objective 2.

Variable	N	Mean	SD	Min	Max
Burnout Parental	561	34.58	33.11	0	137
Co-parent Cooperation	549	17.38	3.76	4	20

### Inferential statisticians

A linear regression model was estimated to determine the relationship between Parental Burnout and families with children under 4 years of age. For this model, the following control variables were considered: Age of children, gender of parents, remote work (at least one parent working remotely), and individual overload (if the overload level is felt by respondent or by their partner). This model explains 6% of the parental burnout variance (R2 = 0.61). It is observed that in families where there are children between 0 and 4 years at home, 5.59 more points are obtained on the Parental Burnout scale than in families having children between 5 and 18 years (*B* = 5.59, *p* < 0.01). Mothers have 15.27 units more of parental exhaustion than their partners after controlling by covariates (B ≈ 15.27, *p* < 0.000), while if the partner works remotely the respondent presents 6.32 units of parental burnout after controlling by covariates (B ≈ 6.32, p ≈ 0.02; see Tables 3, 4).

**Table 3:** Objective regression model 1 with multiple imputations.

Coefficient	*B*	se	*p*
(Intercept)	15.6	5.99	0.01^**^
Children between 0 and 4 years old	5.59	2.15	0.01^**^
Children between 5 and 9 years old	1.45	1.97	0.45
Children between 10 and 14 years old	2.29	2.27	0.31
Children between 15 and 18 years old	−1.13	2.23	0.61
Gender Mother	15.2	3.59	0.001^***^
Individual Remote Work (yes)	−6.75	3.58	0.06
Remote Work Couple (yes)	6.32	2.90	0.05^*^
Individual Overload	1.63	1.92	0.39

* *p* = 0.05;

** *p* = 0.01; and

*** *p* = 0.001

**Table 4:** Objective regression model 2, without multiple imputations.

Coefficient	*B*	se	*p*
(Intercept)	34.6	9.68	0.001^***^
Children between 0 and 4 years old	33.3	10.7	0.01^**^
Children between 5 and 9 years old	1.73	2.12	0.41
Children between 10 and 14 years old	1.79	2.48	0.46
Children between 15 and 18 years old	−2.33	2.32	0.31
Gender Mother	13.7	3.39	0.001^***^
Individual Remote Work (yes)	−3.63	3.95	0.35
Remote Work Couple (yes)	7.94	3.33	0.01^**^
Co-parent cooperation	−0.91	0.48	0.05^*^
Children between 0 and 4 years old X Co-parent cooperation	−1.57	0.58	0.01^**^

* *p* = 0.05;

** *p* = 0.01, and

*** *p* = 0.001.

**Figure 1:**
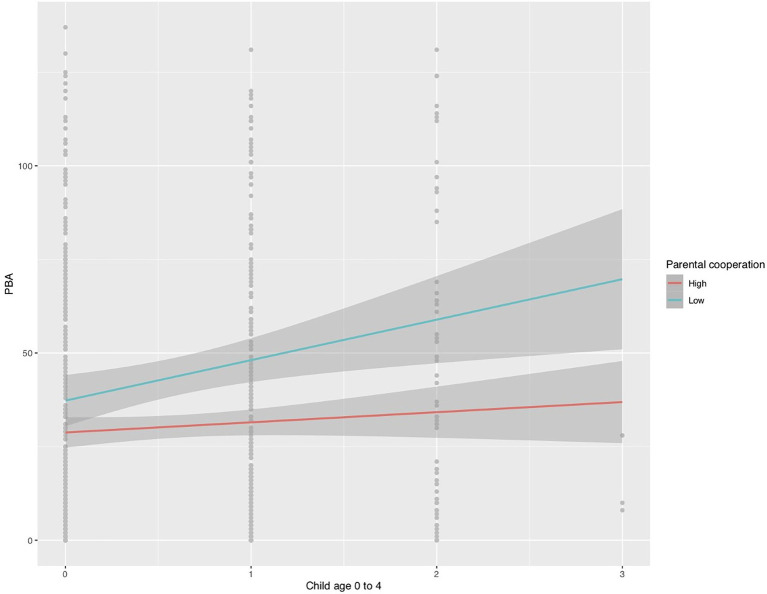
Moderation of the PB in the face of co-parent cooperation. Greater coparental cooperation (red line) results in lower PBA scores for parents with children aged 0–4 years compared to parents with low co parental cooperation (blue line).

To determine if having children under 4 years of age moderates the relationship between co-parent cooperation and Parental Burnout, a linear regression model was also estimated, using the following control variables: age of children, co-parent cooperation score, gender of parents, if any member of the couple or the respondent worked remotely, and the correlation between having children between 0 and 4 years and the score of co-parent cooperation. This model explains 10% of the variance of parental burnout (R2 = 0.10). Concerning the question, it is observed that having children between 0 and 4 years of age moderates the relationship between co-parent cooperation and parental burnout (*B* = −1.57, *p* = 0.01; see Figure 1). As shown in Figure 1, parents who have children between 0 and 4 years old and who present high levels of parental cooperation get lower scores on the parental burnout scale than those who present low levels of coparental cooperation. In relation to couples with remote work, the respondent declares to get about 8 more points in the burnout scale than those who are not in remote working (*B* = 7.94, *p* < 0.01).

## Discussion

This study aimed at determining if PB levels are higher in families with at least one child under 4 years of age, and to analyze whether the age of the child moderates the relationship between co-parent cooperation and PB.

### The age of the children matters

Regarding objective one, it was observed that the age of children is a predictor factor against PB. In families with at least one child under 4 years of age, higher levels of PB are observed. While this relationship was present in previous studies, it did not significantly predict PB (Mikolajczak et al., 2021). This suggests that while an under 4 year old child’s autonomy level requires greater supervision by their caregivers, the demands placed in the current context of a pandemic in a household are stronger than the resources parents have available to cope with them. This makes sense when considering the findings of an earlier study presented by the same team of researchers (Escobar et al., in press), where children at younger ages presented more significant disruptive behaviors and low tolerance to frustration, which is expected due to their level of emotional development (Bonanno and Mancini, 2008; Ashikkali et al., 2020), but at the same time it is more demanding for parents to address.

Given the fact that children younger than 4 years present low levels of brain maturity, their main caregivers are key to regulating stress (National Scientific Council on the Developing Child, 2020). Therefore, a stressful context such as a pandemic makes possible a more insistent search for its “secure base” (Hostinar et al., 2014; Yirmiya et al., 2020; Yoshida and Funato, 2021) as an adaptive response to a high stress-level environment. This caused bigger difficulties within families during the isolation period, since there was an increasing demand from the side of children, together with a lack of external support for daily care (for example the closure of kindergartens, or the lack of adult caregivers to support them in raising their children, etc.).

In terms of the number of children, the results confirm what previous studies had already found (Le Vigouroux and Scola, 2018; Mikolajczak et al., 2018b), where it does not appear to cause a significant effect on PB. These results reinforce the hypothesis that it is the lack of autonomy from the little ones which makes parenting tasks more demanding.

The changes in routines experienced by young children during the pandemic demanded more attention, physical and emotional efforts from their parents (Wei et al., 2022), in addition to the difficulty of reconciling the routines of remote work with those of care and home, what may be the sources that led to greater PB, especially in the mothers who took part in our study. Previous evidence shows gender inequalities in parenting and domestic chores (Goverment of Chile, 2020) and the way this is reflected in PB scores, corresponding to national and international studies. (Kawamoto et al., 2018; Le Vigouroux and Scola, 2018; Pérez-Díaz and Oyarce Cádiz, 2020; Roskam et al., 2021). The result found in mothers who have suffered more PB compared to fathers is a confirmation of what was observed prior to COVID-19 (Pérez-Díaz and Oyarce Cádiz, 2020), but also emphasizes the urgency of considering care policies oriented to women, who have been the most affected during the pandemic (Alon et al., 2020; Mangiavacchi et al., 2021).

### Coparental cooperation and PB

With regard to the second objective, it was found that having children between 0 and 4 years of age moderates the relationship between coparental cooperation and PB. The fact that we found a moderation in terms of co-parenting cooperation is relevant, given that it is teaching us that mothers of children between 0 and 4 years old are the ones who would benefit the most in terms of a more equitable distribution of domestic and parenting tasks. Considering the impact of co-parenting, and the way it would benefit mothers, it is relevant to estimate what it could mean for children. Studies show that the quality of parenting is linked to better acquisition of inhibitory control as well as lower impulsivity in children (Teubert and Pinquart, 2011; Altenburger and Schoppe-Sullivan, 2021), predicting social and emotional adjustments, which could become a protective factor against possible future pandemics or natural catastrophes.

Lastly, the following findings should be considered when making public policy decisions. Families with children under 4 years of age, where the couple is in remote work and who answered the survey, declared to have suffered more PB than couples who did not remote work. From this result, we can state that it has become urgent to rethink the flexibility of work and carry out measures that foster a better balance within families with young children, flexibility that, given the above, should benefit both, mothers and fathers. In this line, it is impressive to see that in families where the responding partner performs remote work, the risk of suffering more PB units is higher in both, the objective one sample and objective two. Contrary to what might be thought, the fact that the partner is working remotely implies for the respondent one a significant source of stress, what leads us to reflect on the roles played in a family by those who care for and are in charge of the domestic tasks of the household, and who in turn are the ones who carry the mental burden of the activities. Therefore, putting the focus on how key coparental cooperation would be for families in future situations involving confinement is a priority. Another impressive finding that turns up a tendency in our data is that the remote work modality was protective for the objective one sample due to the existence of facilitating mechanisms for caregivers, especially those caring for young children, so that being able to balance the difficult work-family roles will continue to be an excellent measure of caregivers’ emotional wellbeing.

### Implications

Evidence indicates that when the pandemic arose, arents perceived an increase in their chronic stress levels (Brown et al., 2020; Garbe et al., 2020; Goldberg et al., 2020; Aguiar et al., 2021; Calvano et al., 2021; Spinelli et al., 2021). This increase is reflected in higher levels of capillary cortisol (Perry et al., 2022), higher scores of Parental Burnout (Bastiaansen et al., 2021), higher risk of neglect and maltreatment in children whose parents presented a greater imbalance between their demands and resources (Griffith, 2020). It is important to highlight that the current study taught us the following: while these factors are common in parents, parents of children at younger ages are at higher risk of PB, which makes this finding important in two ways: firstly, it alerts us about the chronic stress that caregivers of young children are going through, and secondly it works as a wake-up call about how this may impact on the mental health of younger children, as parents under chronic stress such as PB are less likely to be involved, more irritable and distant, what affects interactions with their children in a negative way, therefore, thinking of new strategies for parenting support, and carrying out measures that facilitate work/parenting for both fathers and mothers have become a priority. In addition, it is necessary to promote public policies that foster co-parental cooperation, particularly in families with children at earlier ages.

### Limitations and future research

This work owns limitations that must be considered when generalizing the results. We accessed our sample through social networks and the internet, since a large percentage of our sample comes from higher socioeconomic levels and have achieved higher levels of education (undergraduate and postgraduate studies). This is the reason why, being more generalizable to parts of the population with higher socioeconomic levels and higher levels of education, it is not representative of Chilean families. Currently, in Chile there are 2.1 million people living in poverty, where 831,000 are close to extreme poverty, 15% of children between 0 and 3 years old live in poverty, and 4.3% live in extreme poverty (Ministerio de Desarrollo Social, 2019).

This piece of information must be considered as different studies have proved socioeconomic variables play a key role when foreseeing the mental health of families in contexts of crisis and pandemic (Fonseca et al., 2016; Glynn et al., 2021) where a large percentage of families may be invisible in our study, mainly those with fewer economic and educational resources. Future studies will be able to expand on the findings of this study by using samples of lower socioeconomic status.

To conclude, the use of social networks and the Internet, mainly focused on psychology and parenting, occurred because of the level of education and socioeconomic status, which was common in studied developed in Chile during the COVID-19 pandemic (Dagnino et al., 2020; Larraguibel et al., 2021), but also due to the high participation of women over men in such studies. New strategies are needed to involve more men in parenting studies.

## Data availability statement

The raw data supporting the conclusions of this article will be made available by the authors, without undue reservation.

## Ethics statement

The studies involving human participants were reviewed and approved by Ethics Committee of three universities, the Adolfo Ibáñez University (23/2020), the Pontifical Catholic University of Chile (200425001), and the University of Tilburg in the Netherlands (EC-2018.13). The patients/participants provided their written informed consent to participate in this study.

## Author contributions

CP participated in analysis, in writing the manuscript and in giving final approval. ME participated in the development of the study, in data collection work, editing and made important contributions to the manuscript and in giving final approval. EF participated in analysis. MS and DO participated in data collection work and made final comments. All authors contributed to the article and approved the submitted version.

## Funding

This work was supported by grant a from the Chilean National Fund for Scientific and Technological Development (FONDECYT, grant no. 11190565), by the Early Adversity and Abuse Research Center, CUIDA, and the ANID Millennium Science Initiative/Millennium Institute for Research on Depression and Personality-MIDAP ICS13_005. And CPG was financially supported by the doctoral scholarship program ANID folio 21220467.

## Conflict of interest

The authors declare that the research was conducted in the absence of any commercial or financial relationships that could be construed as a potential conflict of interest.

## Publisher’s note

All claims expressed in this article are solely those of the authors and do not necessarily represent those of their affiliated organizations, or those of the publisher, the editors and the reviewers. Any product that may be evaluated in this article, or claim that may be made by its manufacturer, is not guaranteed or endorsed by the publisher.

## Figures and Tables

**Table:** 

Variable	Level	*N*	%
**Objective 1**			
Gender	Father	525	80.65%
	Mother	126	19.35%
Family type	Biparental	472	72.50%
	Single parent	90	13.82%
	Homoparental	5	0.77%
	Family reunited	46	7.07%
	Multigenerational	30	4.61%
	Polygami	3	0.46%
	Other	5	0.77%
Family income	Less than 200,000	3	0.46%
	Enter 201.000 and 350.000	21	3.23%
	Enter 350.001–500.000	25	3.84%
	Enter 500.001–800.000	41	6.30%
	Enter 800.001 and 1.200.000	74	11.37%
	Enter 1.200.001 and 2.000.000	112	17.20%
	Over 2,000,000	366	56.22%
Educational level	Incomplete basic education	2	0.31%
	Incomplete average	8	1.24%
	Complete media or incomplete technique	26	4.04%
	Incomplete or complete technical University	59	9.16%
	Complete university	271	42.08%
	Postgraduate (Master, Doctor, or equivalent)	277	43.01%
**Objective 2**			
Gender	Father	122	21.75%
	Mother	439	78.25%
Family type	Biparental	472	72.50%
	Homoparental	5	0.77%
	Family reunited	46	7.07%
	Multigenerational	30	4.61%
	Polygami	3	0.46%
	Other	5	0.77%
Family income	Less than 200,000	3	0.53%
	Enter 201.000 and 350.000	15	2.67%
	Enter 350.001–500.000	14	2.50%
	Enter 500.001–800.000	28	4.99%
	Enter 800.001 and 1.200.000	56	9.98%
	Enter 1.200.001 and 2.000.000	100	17.83%
	Over 2,000,000	336	59.89%
Educational level	Incomplete basic education	2	0.36%
	Incomplete high school education	7	1.26%
	Complete high school or incomplete technical career	17	3.05%
	Incomplete university or complete technical career	44	7.90%
	Complete university	238	42.73%
	Postgraduate (Master, Doctor, or equivalent)	248	44.52%

## Appendix 1

Characteristics of the participants.
